# Does COVID-19 Affect the Exercise Capacity of Non-hospitalized Patients?

**DOI:** 10.7759/cureus.18135

**Published:** 2021-09-20

**Authors:** Guillermo A Mazzucco, Rodrigo Torres-Castro, Leonardo Intelangelo, Belen Vila Ortiz, Ana Lista-Paz

**Affiliations:** 1 Unidad de Investigación en Kinesiología Cardiorrespiratoria – Centro Universitario de Asistencia, Docencia e Investigación, Universidad del Gran Rosario, Rosario, ARG; 2 Unidad de Rehabilitación Cardiopulmonar, Instituto Cardiovascular de Rosario, Rosario, ARG; 3 Departamento de Kinesiología, Facultad de Medicina, Universidad de Chile, Santiago, CHL; 4 PhysioEvidence, International Physiotherapy Research Network, Barcelona, ESP; 5 Unidad de Investigación Musculoesquelética – Centro Universitario de Asistencia, Docencia e Investigación, Universidad del Gran Rosario, Rosario, ARG; 6 Departamento de Cardiologia, Instituto Cardiovascular de Rosario, Rosario, ARG; 7 Facultad de Fisioterapia, Universidade da Coruña, A Coruña, ESP

**Keywords:** covid-19, sars-cov-2, exercise capacity, oxygen consumption, functional capacity

## Abstract

Objective

To determine whether non-hospitalized adults post COVID-19 have impaired exercise capacity.

Design

Retrospective analysis.

Setting

Cardiovascular outpatients unit in Instituto Cardiovascular de Rosario, Argentina.

Patients

Eighty non-hospitalized patients post-infection by COVID-19.

Interventions

Participants completed an ergometry pre and post COVID-19 infection.

Main outcome measures

The study's main variables were the metabolic equivalents of task (METs) and the indirect peak oxygen consumption (VO_2 _peak).

Results

The median of METs was 11.7 (9.4-14.8) and 11.7 (11-11.7) in pre and post ergometry, respectively, (p = 0.022). The median VO_2_ (mL/Kg/min) was 21857 (16938-32761) and 21699 (17004-26467) in pre and post ergometry, respectively, without significant differences.

Conclusions

We found slight differences in maximal physical capacity evaluated through exercise testing in non-hospitalized patients by COVID-19.

## Introduction

In recent months, there has been increasing evidence about the respiratory and cardiovascular alterations, with a subsequent impairment of exercise capacity, in patients hospitalized for coronavirus disease 2019 (COVID-19) [1]. However, in people not hospitalized, there is still little information. It has been shown that non-hospitalized patients also have persistence of respiratory symptoms [2]. A recent study that followed 180 non-hospitalized patients found that 53% had symptoms four months after discharge, with around 30% of patients complaining of persistent fatigue [2]. Additionally, it has been reported that many of these patients can have chest tomography-confirmed diagnoses of pneumonia without hospitalization requirements [3]. These findings, added to the mandatory quarantine process during the disease, make us wonder whether exercise capacity is affected in non-hospitalized patients by COVID-19.

The exercise capacity reflects multiple organ systems’ integrated functions, such as respiratory, cardiovascular, and/or musculoskeletal [4]. As such, it is an essential measure of overall health and the body's ability to respond to internal and external stressors, such as COVID-19 [4]. The literature has shown that maximal exercise capacity obtained from an exercise stress test before severe acute respiratory syndrome coronavirus 2 (SARS-CoV-2) infection has been independently and inversely associated with the likelihood of hospitalization due to COVID-19 [5].

Given that hospitalized patients' information cannot be extrapolated to non-hospitalized patients, we aimed to determine whether non-hospitalized adults post-COVID-19 have impaired exercise capacity.

## Materials and methods

A retrospective study was conducted at the Instituto Cardiovascular de Rosario (ICR), Argentina, including a) people over 18 years; b) non-hospitalized patients; c) diagnosis confirmed by positive reverse transcription-polymerase chain reaction (RT-PCR); d) an exercise stress test on a treadmill in the past three years and eight months within the ICR and a recent stress test post-SARS-CoV-2 infection [5].

All the patients who performed a post-COVID-19 infection stress test were consecutively added to a database; then we analyzed which patients had a stress test performed in the ICR no greater than 3.8 years before the infection. Only the most recent test was included when more than one test was available for a given patient. Before the test, the cardiologist investigated cardiovascular risk factors and the current symptoms related to COVID-19 and recorded the data in the electronic medical record. The ergometries were performed by trained cardiologists mostly using the modified Bruce protocol and according to the American Heart Association guidelines [6]. Patients were asked to give their best effort, and tests were stopped following signs or symptoms or patient exhaustion. The study's main variable was the metabolic equivalents of task (METs), calculated by the software (ErgoView, Eccosur, Argentina) based on the speed and degrees of inclination of each stage. Additionally, the indirect peak oxygen consumption (VO2 peak) was estimated [7].

Our institutional board approved this study. This study did not require informed consent, given the retrospective nature of the design. For data analysis, an anonymized database was developed and used.

The results were reported as mean ± SD or median and interquartile range (q1-q3) for continuous variables after testing their normal distribution with the Shapiro-Wilk test. The categorical variables were expressed as absolute and relative frequencies. Paired t-test or Wilcoxon signed-rank test was performed to assess differences between continuous data before and after COVID-19. McNemar's test was performed to assess differences between categorical data. Statistical significance was established in p < 0.05. All statistical analyses were performed in SPSS version 26.0 (IBM Corporation, Armonk, NY).

## Results

After the initial screening of the database of 299 patients, 80 met the selection criteria (47.1 ± 12.2 years, 50% women). Among the comorbidities, the following stood out: dyslipidemia (27.5%), arterial hypertension (20%), and obesity (15%). The median elapsed between pre and post-ergometry was 1.7 (1.2-2.5) years, and the time elapsed between the discharge and ergometry was 56.5±26.6 days. At least one symptom post-COVID-19 was present in 25% of the sample, mainly dyspnea (n = 14) and fatigue (n = 9). Complete sample characteristics are shown in Table 1.

**Table 1:** Characteristics of the participants (n = 80). Data are expressed as n (%) unless otherwise stated.

Females	40 (50)	
Age (years) mean (SD)	47.1 ± 12.2	
BMI (kg/m^2^) median (q1-q3)	25.3 (22.6-28.6)	
Smoking habit (never/former/current smoker)	59 (73.8)/ 17 (21.3)/ 4 (5)	
Family history of cardiovascular diseases	17 (21.3)	
Comorbidities		
Dyslipidemia	22 (27.5)	
Hypertension	16 (20)	
Obesity	12 (15)	
Diabetes	5 (6.3)	
Years between pre and post-ergometry median (q1-q3)	1.7 (1.2-2.5)	

The median of METs was 11.7 (9.4-14.8) and 11.7 (11-11.7) in pre and post-ergometry, respectively, (p = 0.022). The median VO2 (mL/Kg/min) was 21857 (16938-32761) and 21699 (17004-26467) in pre and post-ergometry, respectively, without significant differences. The resume of the other parameters is included in Table 2.

**Table 2:** Comparison between pre and post-COVID-19 ergometries (n = 80). Data are expressed as mean (SD) unless otherwise stated Paired t-test or Wilcoxon signed-rank test was performed to assess differences between continuous data. McNemar's test was performed to assess differences between categorical data. HR: Heart rate; VO2: Oxygen consumption. b VO2 was indirectly calculated with equations proposed by the American College of Sports Medicine [7].

	Pre-COVID-19 Ergometry	Post-COVID-19 Ergometry			
Negative/Positive ergometry n (%)	79 (98.8)/1 (1.3)	74 (92.5)/6 (7.5)	.125		
Speed (km/h) median (q1-q3)	6.7 (5.4-8)	6.7 (6.6-6.7)	.754		
Ramp (º) median (q1-q3)	15 (14-18)	15 (14-15)	.010*		
METs median (q1-q3)	11.7 (9.4-14.8)	11.7 (11-11.7)	.022*		
VO_2_ (mL/kg/min)^b^ median (q1-q3)	21857.2 (16938.3-32761.1)	21699.8 (17004.1-26467.4)	.220		
Basal HR (bpm)	78.4 ± 13.9	80.8 ± 13.8	.146		
Maximum HR (bpm)	167.9 ± 16.1	168.4 ± 17	.444		
Recovery HR min 1	139.3 ± 16.7	139.3 ± 18.9	.988		
Recovery HR min 2	124.7 ± 15.6	120.2 ± 18.5	.017*		
Recovery HR min 3	109.7 ± 15.3	107 ± 17.3	.164		

## Discussion

In this series of 80 non-hospitalized patients by COVID-19, we found slight differences in METs evaluated through exercise testing and no differences in the estimated VO2 peak. This minimal difference is also toward improvement, showing no deleterious effects of COVID-19 in non-hospitalized patients.

Brawner CA et al. analyzed 89 hospitalized vs. 157 non-hospitalized patients and found that maximal exercise capacity was independently and inversely associated with the likelihood of hospitalization due to COVID-19. Despite the significant number of patients, the authors did not analyze the non-hospitalized patients. Interestingly, they showed that the hospitalization rate in patients with METs >9.7 was lower than 30%. Although our patients are younger than Brawner’s patients, these results are in line with our results because the median of METs of our patients was 11.7, confirming a possible protective role of the pre-infection maximal capacity exercise for hospitalization [5]. 

In a retrospective study, Jacobson KB et al., assessed 118 non-hospitalized patients (mean age of 43.3 ± 14.4 years), in a four-month follow-up after the initial COVID-19 diagnosis. They showed that patients walked 60% of the predicted value during the Six-Minute Walk Test (6MWT). The 6MWT measures submaximal physical capacity, so it is not comparable with the ergometry result that measures maximum capacity [8]. Another probable cause of the differences is that the persistence of at least one symptom in the Jacobson et al. population was 64.2% (more than double our prevalence).

In another study evaluating the effect of SARS-CoV-2 in the Swiss military, with post-ergometry close to two months after discharge, similar to our data, they found that physical ability was unchanged in most non-convalescent patients [9]. Our results are in line with the Crameri study, although this population is notably younger (20.8, 19.9-21.9 years) and more trained. However, it is interesting that the results of trained subjects are similar to our non-trained patients [9].

Our report has several strengths. So far, there are very few studies with stress testing in post-COVID-19 patients. We have the pre and post-infection stress tests for COVID-19 with a reasonable range of time between them and following the international recommendations for assessing physical capacity [10]. Finally, we included non-hospitalized patients since most of the studies have focused on the hospitalized population. Additionally, our study is not without limitations. The nature of the study is retrospective and does not have a control group. However, we have the advantage of having a previous test. On the other hand, we have a small sample of patients, only a few with persistent symptoms, indicating a possible explanation for affected physical capacity.

## Conclusions

We found slight differences in maximal physical capacity evaluated through exercise testing in non-hospitalized patients by COVID-19. Considering the possible differences between non-hospitalized versus hospitalized patients, it is necessary that future studies explore the maximal physical capacity in non-hospitalized patients, particularly in those who present persistent symptoms after COVID-19.
